# Flint on Continued Fever

**Published:** 1852-09

**Authors:** 


### FLINT ON CONTINUED FEVER.

Through the *Buffalo Medical Journal* we had been made acquainted with most of the matter of this work, which consists of three reports on Continued Fever, based on the analysis of one hundred and sixty-four cases treated by Professor Flint at the Buffalo Hospital of the Sisters of Charity. The labor of preparing these reports was undertaken at the request of the New York State Medical Society, with particular reference to the question of the identity or non-identity of Typhus and Typhoid fever. The cases observed by the author since the assignment to him of this duty, he has subjected to numerical analysis, and the results are here embodied in a form highly creditable to our medical literature. He is very safe in indulging the hope, that his work "may be thought by those whose approbation he would be most ambitious to obtain, to contribute somewhat to the study of the natural history of the several forms of continued fever, and to the aggregation of facts having important bearings on diagnosis, pathology and therapeutics." His statistical details possess uncommon interest, and in his use of them he shows a mind at once cautious, logical and comprehensive. The reader would search in vain through the volume for one single instance of hasty deduction. All is fair, unprejudiced, calm, and philosophical. The author had no cherished theory to sustain by bending facts to suit it, and when it happened that he held an opinion which his inquiries did not sanction he unhesitatingly abandoned it. Thus, he set out a believer in the identity of the fevers in question, but before he completed his reports he announces that "the study and thought bestowed upon the subject" had led to a change in his views. As a specimen of cautious induction, the work is worthy of study, especially in our profession, which has abounded more than most others in examples of loose, inconclusive reasoning. The style in which it is composed is also admirable for a scientific work. It is quite evident that Dr. Flint has not regarded style as a matter to be entirely overlooked even in such a work, and that he has cultivated it with great success. One is not only satisfied as he proceeds with the fairness of his argument, and instructed by his full, lucid details, but pleased and gratified by the easy, natural, graceful flow of his language.

In connection with these reports, Dr. Flint treats of the identity of Typhus and Typhoid fever; the diagnosis differential of the two affections; the discrimination of continued fevers from other diseases; the management of continued fever; the morbid appearances after death; relapsing fever; and the transportation and diffusion, by contagion, of Typhoid fever, exemplified in its occurrence at North Boston, Erie county, New York. As the language of the last member of this sentence implies, he believes in the contagiousness of the disease. He thus forcibly puts the case :—

"In a small, isolated community, consisting of nine families, seven of which lived within a few rods of each other, a person is introduced affected with *Typhoid* fever, and after lingering twenty-nine days, dies with this disease. Prior to that event the members of this community were free from disease of any kind—the situation was in every respect healthy, and *Typhoid* fever was a disease unknown in that place and neighborhood. The patient lingered and died at the public house, which was a place of daily resort for the members of those seven families with a single exception. One family, consisting of several persons, living but four rods from the tavern, was on terms of hostility with the inn-keeper which precluded all intercourse. The arrival of a sick stranger with a severe disease was an event of interest to the inhabitants. He was visited, more or less, daily by the different members of the families living closely at hand, with the exception of the family referred to; and the family of the inn-keeper were of course brought into close contact with the disease. Twenty three days after the arrival of the stranger, two members of the family of the inn-keeper were attacked with the disease, and subsequently five others in his family. In all the other families living within a few rods of the tavern, excepting a single family, cases occurred, more or less in number, within the space of about a month from the date of the first case developed after the stranger arrived; and during this period more than half the population of the settlement were affected. The disease then ceased further progress, no cases afterward occurring. The family in which no cases occurred was the only family of the seven not brought into contact with the disease. The relations of this family to that of the inn keeper, as just stated, precluded all social intercourse, and shortly after the disease began to spread, its production being imputed to the agency of this family, all intercourse with the other families affected with the disease was at once suspended.

"Now in view of this reviewal of the facts, if it be claimed that the disease was not transported to the place and diffused by contagion, it is necessary to admit a series of coincidences almost incredible. It will be seen that the circumstances which have just been rehearsed, embrace all those which had been previously mentioned as important conditions for a fair experiment in order to test the contagiousness of a disease. In fact, if every circumstance connected with the history of the disease at North Boston had been deliberately selected and arranged for a scientific object, they could hardly have been rendered more complete or judicious."

Dr. Flint closes his remarks on the management of continued fever, with a sentiment so just and important, that we are glad to have an opportunity of reproducing it for the benefit of our readers:—

"There is one point, pertaining to diet, respecting which the views commonly entertained appear to me to be faulty. I refer to the apprehensions frequently, if not generally felt by practitioners, in allowing a nutritious animal diet early in convalescence. These apprehensions, which I am persuaded are imaginary, have probably arisen, from the habit of associating Inflammation with Fever. The susceptibility to disorder, or consecutive diseases, is probably owing to the enfeebled condition of the organism after passing through the febrile career. It is an object, therefore, in the management of convalescence, to effect a return of the accustomed constitutional vigor as speedily as practicable. The means for this end are, aliment of the most digestible and nutritious kind, tonics, stimulants, and hygienic measures, of which one of the most useful is early gestation in the open air. I have pursued this course in a sufficient number of cases to feel satisfied from experience that it is not only unattended by danger, but is conducive to the well being of the patient. I have never witnessed any unpleasant results from the effort to re establish the strength of the patient by resorting at once to an animal diet, etc."

We received this work too late for a full notice of its contents in our present number. From what we have said, our readers will gather that our opinion of it is a very favorable one. We believe that it will take a high rank among the professional works of our country, and a permanent place in the literature of continued fever. It is a model for such researches, and in this light will be most highly appreciated by those who have had most experience and are most skilled in the investigation of disease. It is very carefully printed, and forms a neat octavo volume of 398 pages. The publishers are H. Derby & Co., Buffalo. We are indebted for a copy of it to T. R. Nelson, of this city.

